# Compositional equivalence assessment of insect-resistant genetically modified rice using multiple statistical analyses

**DOI:** 10.1080/21645698.2021.1893624

**Published:** 2021-03-01

**Authors:** Seon-Woo Oh, Eun-Ha Kim, So-Young Lee, Da-Young Baek, Sang-Gu Lee, Hyeon-Jung Kang, Young-Soo Chung, Soon-Ki Park, Tae-Hun Ryu

**Affiliations:** a R&D Coordination Division, Rural Development Administration, Jeollabuk-do, Republic of Korea; b National Institute of Agricultural Sciences, Rural Development Administration, Jeollabuk-do, Republic of Korea; c Department of Molecular Genetic Engineering, Dong-A University, Busan, Republic of Korea; d School of Applied Biosciences, Kyungpook National University, Daegu, Republic of Korea

**Keywords:** Rice (*Oryza sativa* ssp. *japonica*), Bt rice, GMO, Natural variation, Composition

## Abstract

The safety of transgenic Bt rice containing bacteria-derived *mCry1Ac* gene from *Bacillus thuringiensis* (Bt) was assessed by conducting field trials at two locations for two consecutive years in South Korea, using the near-isogenic line comparator rice cultivar (‘Ilmi’, non-Bt rice) and four commercial cultivars as references. Compositional analyses included measurement of proximates, minerals, amino acids, fatty acids, vitamins, and antinutrients. Significant differences between Bt rice and non-Bt rice were detected; however, all differences were within the reference range. The statistical analyses, including analysis of % variability, analysis of similarities (ANOISM), similarity percentage (SIMPER) analysis, and permutational multivariate analysis of variance (PERMANOVA) were performed to study factors contributing to compositional variability. The multivariate analyses revealed that environmental factors more influenced rice components’ variability than by genetic factors. This approach was shown to be a powerful method to provide meaningful evaluations between Bt rice and its comparators. In this study, Bt rice was proved to be compositionally equivalent to conventional rice varieties through multiple statistical methods.

## Introduction

The biosafety of genetically modified (GM) crops is generally determined via a safety evaluation assessment before commercialization. Assessment regulations differ by country but are all implemented based on risk assessment according to their substantial equivalence to non-GM commercial crops.^1,2,1^ Comparative compositional studies for the safety assessment of food and feed from GM crops are typically conducted on a crop-specific basis according to the principles outlined in the Organization for Economic Co-operation and Development (OECD). Analytes representing key nutrients, antinutrients, toxicants, and secondary metabolites from GM crops are compared to data collected from non-GM conventional counterparts. The comparative safety assessment approach based on the concept of substantial equivalence assures that foods and feeds derived from GM crops are as safe as their conventional counterparts.^3^ Although the application of the concept is not a safety assessment in itself, it leads to identifying potential differences between the GM crops and the conventional crops and to further investigate the safety assessment with respect to their toxicological impact. Besides, it needs to recognize some of the limitations of the concept of substantial equivalence in GM food risk assessment. Kuiper et al.^4^ have reported practical difficulties may encounter for the application of the principle in particular respect to the availability of the direct comparator, limited information on the appropriate safe conventional comparators in levels of relevant crop constituents, and limited availability of methods for the detection and characterization of unintended effects. Natural variation of nutrients and antinutrients in plant cultivars may vary randomly and cause no harm to human health. However, continuously lower or higher concentration may result in safety concerns despite its natural variation.^4^ To increase the chances of detecting unintended effects, non-targeted profiling techniques such as transcriptomics, proteomics, and metabolomics have been encouraged to be included.^4–8^

A significant difference in the amounts of nutritional analytes in GM crops compared to those of their conventional comparators needs to be evaluated within the natural variation of conventional crop components that already possess a history of safe use as foods.^3^ It is recommended to cultivate conventional non-transgenic commercial varieties (termed reference) concurrently at the same sites as the GM crop and its direct counterpart in order to obtain an overview of the impact of genetic variation on compositional variability.^8,9^ The reference data of conventional crops is also available from the OECD, the ILSI Research Foundation Crop Composition Database (ILSI-CCDB), or from published literature. The evaluation of natural variability in the composition of conventional crops and GM crops has been incorporated into their compositional assessment in several studies, using statistical approaches.^10–15^ Recently, the empirical distribution curves of compositional end-points for maize and soybean were determined, providing novel information on end-point specific variability relevant to risk assessment.^16^

Rice is one of the major staple crops and primary food sources of more than half of the world’s population. Multiple transgenic rice plants with the insecticidal Cry proteins from the bacterium *Bacillus thuringiensis* (Bt) have been developed to control harmful insects, improve crop productivity, and benefit the environment.^17–23^ In South Korea, several Bt transgenic rice lines that are expressing Cry1 proteins have been developed.^21,24–26^ Bt 9 rice contains mCry1Ac1, modified from the original Bt Cry1Ac1 by deleting the C-terminus (Genebank No. AY126450) for insect resistance and *bar* (which confers herbicide tolerance) as a selection marker.^26^ It was suggested Bt rice products are compositionally equivalent to their non-transgenic counterparts.^14,15^ Recently, Liu et al. showed that unintended changes of Bt rice do not go beyond conventional cross-breeding using the approaches of transcriptomics and metabolomics.^16^

In the present study, we employed the substantial equivalence assessment to determine the compositional safety of Bt 9 rice. The analyte levels in the insect-resistant Bt 9 rice were compared with those in its near-isogenic non-Bt rice (direct counterpart). When one or more compositional components in Bt rice significantly differed from those in non-Bt rice, the levels of analytes of concern in Bt rice were compared with those in four non-transgenic commercial rice cultivars (from now on referred to as reference) grown simultaneously with Bt rice and non-Bt rice in two sites for 2 years. In addition, the range of values obtained for all reference rice across sites and years (from now on referred to as all reference), and the OECD literature data^27^ revealed that the analyte levels in Bt rice are similar to those in conventional varieties. We performed multivariate analyses to obtain meaningful data on natural variation among genotypes and variation due to environmental effects and their interactions with genotypes. Multivariate analyses included % variability analysis (variance component analysis), ANOSIM, PERMANOVA, and SIMPER. We suggested that a multi-statistical analysis approach would be useful to develop more reliable and effective methods in GMO safety evaluation.

## Materials and Methods

### Field Trial Samples

Insect-resistant Bt 9 rice (*Oryza sativa* ssp. *japonica* ‘Ilmi’), non-Bt rice (Ilmi), and four non-transgenic commercial rice varieties (Samkwang, Seolhyangchul, Hiami, and Hwaseong) were simultaneously cultivated in 2015 and 2016. Field sites were in Jeonju (35°83´08.57´´N, 127°06´62.29´´ E) and Suwon (37°16´20.02´´N, 126°59´02.85´´ E) where localized in the central and northern regions of South Korea, respectively. Four commercially available rice varieties with similar genetic backgrounds to ‘Japonica’ were used as reference materials for the GM rice safety assessment. They are high-quality rice varieties developed for their desirable characteristics such as rice taste, disease resistance, and grinding characteristics, and then distributed to farmers by the Rural Development Administration of South Korea. All test samples were planted in May in a strip-plot design with five biological replicates per site, and standard commercial agronomic practices were applied at each field site following local practices. Weather conditions, including rainfall, at the cultivation sites, are presented in Table S1 of the Supporting Information. Grain samples from twelve rice plants were pooled to form one biological replicate and then shipped to the laboratory in ambient condition. Grain samples were dried to a moisture concentration of 9–11% in the sun using the plastic sheet for three days. Samples were manually dehulled using a hulling machine (TR 13, South Korea) to produce brown rice grains and subsequently finely ground using a Planetary Mono Mill Pulverisette 6 (Fritsch, Germany). Powdered samples of each rice type were immediately stored at −80°C until subjected to compositional analysis.

### Compositional Analysis Methods

Nutritional components of the analyzed rice powdered samples were proximates (moisture, protein, lipid, crude fiber, ash, soluble dietary fiber (SDF), and insoluble dietary fiber (IDF)), carbohydrates, amino acids, minerals, fatty acids, vitamins, and antinutrients. Three experimental replicates were used per biological replicate, resulting in 15 samples for each analyte.

Moisture content was determined by gravimetric measurements using a hot-air oven set at 105 °C.^28^ The content of nutritional components was calculated on a dry weight basis considering the moisture content. Crude protein content was calculated based on total nitrogen content, using the Kjeldahl method.^29^ Crude fat was analyzed using the Soxhlet extraction method.^30^ Crude fiber was analyzed in an automatic furnace using a filter bag.^31^ For analysis of IDF and SDF analysis, heat stable amylase, protease, and amyloglucosidase were used to dissolve proteins and starches, leaving a fibrous residue.^32^ IDF was determined by weighing a fibrous residue after filtration. For SDF analysis, the samples filtered was precipitated with ethanol and filtered. After dying, the residue weight was determined. Carbohydrate levels were calculated as 100% − (% moisture + % protein + % lipid + % ash).

Sixteen amino acids were directly analyzed using an automatic amino acid analyzer (L-8500-A; Hitachi, Tokyo, Japan), following protein hydrolysis with hydrochloric acid.^33^ The sulfur-containing amino acids cysteine and methionine were oxidized with performic acid before hydrolysis with 6 N hydrochloric acid.^33^ The content of individual amino acids was expressed as a percentage of the total protein. The concentrations of copper, iron, zinc, manganese, calcium, sulfate, magnesium, potassium, phosphorus, and sodium were determined using inductively coupled plasma (ICP) optical emission spectrometry (Inegra XL; GBC Co., Melbourne, Australia).^34,35^ Samples were hydrolyzed in a solution containing sulfuric acid and nitric acid before instrumental analysis of ICP. Fatty acid profiles were determined according to the AOCS method Ce 1–62 using a Shimadzu GC-2010 gas chromatograph (Kyoto, Japan) and expressed as a percentage of total fatty acid.^36^ Fatty acids were extracted with a chloroform: methanol (v/v 2:1) solution containing an internal standard (Pentadecanoic acid solution) and then saponified with toluene, 5 N sodium hydroxide, and methanol. The saponification mixture was methylated with 14% boron trifluoride. The resulting methyl esters were resolved in hexane and analyzed.^36^

Vitamin B_1_ was measured using a modified method of Sims and Shoemaker, using high-performance liquid chromatography (HPLC) analysis.^37^ The samples in 0.1 N HCl were incubated at 80°C for 30 min, and the resulting solution was incubated in a buffered enzyme solution (taka-diastase). The solution was purified on a C_18_ cartridge, and then the filtrate was reacted with potassium ferricyanide to convert thiamine to thiochrome. For vitamin B_2_ analysis, the rice samples were hydrolyzed with 0.1 N HCl, followed by incubation at 80°C for 30 min. The filtered solution using a 0.45 µm syringe filter was analyzed by HPLC fluorometric detection according to the method described by Esteve et al.^38^ For Vitamin B_3_ analysis, the rice samples were autoclaved at 121°C for 15 min in 2.5 N H_2_SO_4_ and followed by solid-phase extraction using a cartridge (Oasis HLB Plus LP Extraction Cartridge). *N*-methyl-*N*-(trimethylsilyl) trifluoroacetamide and pyridine were added to the resulting solution and then incubated with shacking at 60°C for 30 min for derivatization. Each derivatized sample was injected into the Gas-Chromatography/Time-Of-Flight Mass spectrometry (GC Agilent 7890A/TOFMS LECO, MI, USA).^39^ Vitamin B_7_ (Biotin) was determined according to the MFDS Food Code.^40^ The samples suspended in 0.01 M KH_2_PO_4_ were incubated in a sonication bath for 30 min. The suspension was then centrifuged for 30 min at 9,000 × g, and the supernatant was filtered using a 0.45 µm syringe filter before HPLC analysis. The measurement of vitamin E (α-tocopherol) amount was performed as described by Park et al.^21^ Samples were suspended in ethanol containing 0.1% ascorbic acid and incubated at 85°C for 5 min, and then after potassium hydroxide (80%) was added to the suspension, samples further incubated at 85°C for 10 min. 5α-cholestane (internal standard), distilled water, and hexane were added to the chilled suspension. After centrifugation, the upper layer was dried under nitrogen gas. Sample derivatization was performed as described in Vitamin B_3_ analysis, and then vitamin E was analyzed by GC/TOF.^21^

The content of phytic acid was determined as described by Park et al.^21^ The samples were incubated with 2.4% HCl in a sonication bath for 2 hr, and the supernatant was filtered through polyprep-prefilled chromatographic columns (Bio-Rad Laboratories, Richmond, CA, USA) containing an AG-1-X8 anion exchange resin (100–200 mesh chloride form, 0.8 cm × 4 cm). Trypsin inhibitor activity was determined using the AOCS method Ba 12–75.^41^ The rice sample was extracted in 0.01 N sodium hydroxide for 3 hr in a sonication bath, and then varying aliquots of the sample suspension were mixed with a known amount of trypsin and the synthetic substrate, benzoyl-DL-arginine-*p*-nitroanilide (BAPNA). After the mixture was incubated for 10 min at 37°C, the reaction was stopped by the addition of acetic acid. The solution was filtered with a 0.45 µm syringe filter, and then the absorbance was measured at 410 nm.

**Statistical analysis of compositional data**. Statistical analyses of the data were carried out using the SAS 9.2 software package (SAS Institute, Cary, NC, USA). A statistical difference test for Bt rice and non-Bt rice was performed on individual data sets obtained from each site per year, using the independent two samples Student’s *t*-test. In order to identify the effects of genotype and environment (location and cultivation year) on the natural variation in nutritional components, we evaluated the random effect of varieties (G), site (S), cultivation year (Y), and their interactions (G × S × Y) in nutritional variation, using the linear mixed model with random intercepts fitting by restricted maximum likelihood (lme4 package) in R studio (version 4.0.2). It included three variables (Bt, non-Bt, references) for the variety, two variables (Jeonju, Suwon) for the site, and two variables (2015, 2016) for the year. Variance components were estimated with the structure:

Analyte ≈ G + S + Y + GSY + Ɛ

Where analyte is the composition level, G is the genotype, S is the site, Y is the cultivation year, GSY is the genotype × site × year interaction, and Ɛ is the general error term.

The syntax is as follow:

fm1<-lmer(y ~ 1+(1|Site:Variety:Year)+(1|Site)+(1|Variety)+(1|Year), data).

The variance component parameters were divided by the total variance to get the components’ variance proportions. The Plymouth Routines in Multivariate Ecological Research (PRIMER) software package version 6.0 with Add on PERMANOVA (PRIMER-E Ltd, UK) was used for multivariate analysis of variance to identify significant differences among study groups regarding their nutritional profiles, following the method described by Anderson.^42^ Analyses of ANOSIM, PERMANOVA, and SIMPER were performed to define the explanatory power of the variability among the data.^43–45^ Two-way ANOSIM was used to calculate the distance between study groups according to the Bray-Curtis similarity matrix. The Global R-value indicates the similarity among groups, showing a range of −1 to +1. Global R-values close to zero indicate little or no difference among groups, while R-values closer to −1 or +1 indicates significant differences among groups. PERMANOVA utilizes the permutation method to test inter-group differences and examines whether between-group variance explains a significant proportion of the total variance in the system. In this study, PERMANOVA based on 999 permutations using the Euclidean distance and partitioning was done using Type I sum of squares for each factor (G, S, Y, G × S × Y). The significance level of the sample statistic used in PERMNOVA was 0.1%. The SIMPER test was used to determine the average dissimilarity between pairs in the study groups and the contribution (%) of each analyte to the dissimilarity between pairs. It was calculated using the Bray-Curtis matrix, and the cutoff value was 80%.

## Results and Discussion

### Comparative Assessment of Bt Rice and non-Bt Direct Comparator Rice

Compositional analysis results (55 components) for Bt rice and non-Bt rice are presented in Table S2 of the Supporting Information as of the mean value with ± standard deviation (SD), together with their ranges (the minimum and maximum values). The reference ranges (the minimum and maximum values) of each analyte for commercial rice were determined from 20 means of four rice varieties (mean of 5 biological replicates × four varieties) grown simultaneously with Bt rice and non-Bt rice. The all reference ranges (the minimum and maximum values) of each analyte were obtained from the reference ranges across two cultivation sites for two years. Boxplots of component values in Bt rice, non-Bt rice, reference, and all reference are presented in Figure S1-S5.

Overall, a few statistically significant differences between Bt rice and non-Bt rice was characterized (*p* < 0.05). From a total of 220 comparisons, 53 (24.1%) were significant; these included 20 comparisons from Suwon and 33 from Jeonju (statistically relevant differences are highlighted in bold font in Table S2). However, most of the analytes that exhibited significant differences between Bt rice and non-Bt rice were within the ranges of commercial rice varieties planted simultaneously (reference) or were found to be within the ranges reported in the OECD, with the exception of moisture, sulfate, stearate, carbohydrate, and IDF content. Bt rice moisture content was significantly lower (*p* <0 .05) compared with non-Bt rice from both Suwon and Jeonju in 2016. Bt rice moisture content was outside of the reference ranges but were all within the ranges of all reference and of reported in OECD literature. Sulfate and stearate were also significantly lower in Bt rice than in non-Bt rice cultivated in 2015 at Suwon (*p* < 0.05). Sulfate was not within the reference range, but it was within the reported OECD range. Stearate was not within the reference range, but it was within the all reference range and the reported OECD range. For carbohydrates, a statistical difference (*p* < 0.05) was observed between Bt rice and non-Bt rice in 2016 at Jeonju. Bt rice from 2016 Jeonju was not within the ranges of references as well as the OECD range. However, it was within the all reference range. The level of carbohydrate was calculated from 100% − (% moisture + % protein + % lipid + % ash). Since mean values of moisture, protein, lipid, and ash in Bt rice were equivalent to conventional crops, it is reasonable to consider biologically no meaningful difference in carbohydrate. Statistical difference (*p* < 0.05) was observed in the mean IDF content between Bt rice and non-Bt rice in 2016 at Jeonju. Information about the IDF content in brown rice was not available in the OECD literature.^27^ However, the mean IDF content in Bt rice from Jeonju in 2016 was within the all reference range, suggesting that IDF in Bt rice is biologically equivalent to that in conventional crops (Table S2 of the Supporting Information). Taken together, given the paucity of observed differences, the grain of Bt rice was compositionally equivalent to that of conventional comparators.

### Multivariate Analysis

Evaluation of the relative magnitude of natural variation in composition among varieties and variation influenced by environmental factors and their interactions with varieties provides context to the assessment of new GM crops.^15^ Therefore, multivariate statistical analyses were performed to identify the major contributors to the natural variation in nutritional components of the tested rice. The effects of genotype, cultivation site, and cultivation year on nutritional variance were identified by multivariate statistical analyses using percentage variability and variance tests.

**Percentage variability of rice grain nutrients**. Crop compositions depend on genetics and environmental conditions such as cultivar, cultivation site, and year, as well as a management strategy.^46–48^ In order to evaluate natural variation, it is crucial to use extensive data sets obtained from multiple sites and over several years.^46,47^ Recently, Assefa et al. demonstrated that genetics, management strategies, and environmental factors influenced seed composition (protein and oil) and yield in US soybean seeds through meta-analysis and synthesis of a database obtained across the United States of America.^49^ Oh et al. reported that the growing site and cultivation year contributed more to compositional variability in 15 Korean commercial rice varieties through statistical studies.^50^

We performed a percentage variability analysis to determine to what extent components were affected by genotype, site, and cultivation year, as well as their interaction (G × S × Y). This model was previously reported as suitable for field studies by describing the impact of random effects on nutritional variation and determining overall differences and equivalences among samples.^10^

**Figure 1.**
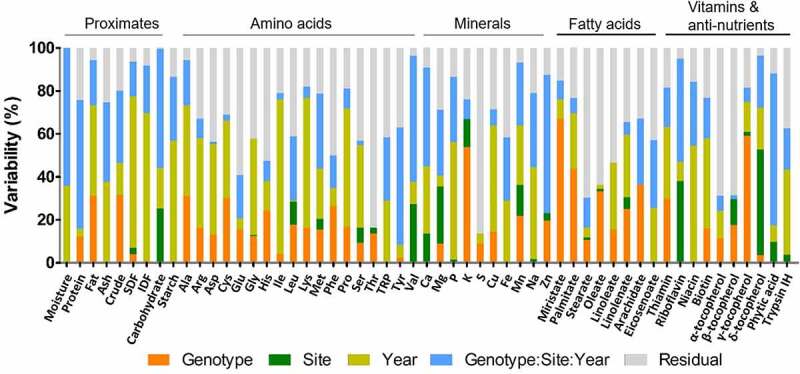
The value of percentage variability for each individual nutritional component in rice grains

The results of percentage variability are presented in Fig. 1. Among the proximate components, SDF, IDF, lipid, and ash contents were highly influenced by cultivation year and accounted for 70.5%, 68.8%, 42.2%, and 37.7% of the total variance, respectively. The G × S × Y effect mainly contributed to the total variance in moisture (64.2%), protein (60%), and carbohydrate (55.4%). The cultivation year (56.9%) and the G × S × Y effect (29.6%) also highly contributed to the total starch content variance. Residuals accounted for relatively high proportions of the total variance in amino acids, with the contents of 11 out of 18 amino acids contributing to more than 30% of the total variance. A large proportion of residual to total variance indicates a problem with a prediction for an observation. It might require more datasets, such as more cultivation years and sites, to lower the residual proportion. Threonine was the highest concentration of amino acids present in the residuals, accounting for 83.7% of the total variance. Cultivation year highly contributed to the total variance in Ile (71.8%), Lys (60.3%), Pro (55%), and Ala (42.2%). Val and Tyr were mainly affected by the G × S × Y effect, accounting for 58.9% and 54.6%, respectively.

Potassium (18.08%) and zinc (14.98%) levels displayed the highest variability, contributed by genotype and site, respectively. The cultivation year and the G × S × Y effect mainly contributed to the total variance for calcium, phosphorus, iron, manganese, and sodium. Residuals determined relatively high proportions of the total variance in fatty acids and contributed to more than 30% of the total variance in all lipids, with the exception of myristate and palmitate. Myristate and palmitate contents were highly determined by genotype, which accounted for 67.2% and 43.7% of the total variance, respectively. Variance in oleate (33.3%) and arachidate (36.5%) was also significantly contributed by genotype.

With respect to the percentage variability in vitamins, niacin (54.7%) and biotin (42.1%) were mainly affected by cultivation year, while riboflavin (48.1%) was influenced by the G × S × Y effect. Cultivation year (33.6%) and genotype (29.8%) contributed to the variation in thiamin in similar proportions, followed by the G × S × Y effect (18.3%). Genotype and cultivation site highly determined the variability of γ-tocopherol and δ-tocopherol, which accounted for 59.1% and 49.2% of the total variance, respectively. However, residuals notably contributed to α-tocopherol and β-tocopherol, accounting for approximately 60% of the total variance. Phytic acid content was highly determined by the G × S × Y effect (70.7%), while cultivation year (39.4%) and residuals (37.5%) contributed to the variance in trypsin inhibitor content. Collectively, the natural variation in rice composition analyzed in the present study was contributed by environmental factors and the G × S × Y effect as well as genotype.

### Variance Analysis Using ANOSIM

Significant differences in cultivation year, site, and genotype among the study groups were further tested using the PRIMER statistical package. ANOSIM and SIMPER are multivariate analyses that use resemblance matrices, with SIMPER decomposing the average Bray-Curtis dissimilarities between all sample pairs.^36^

The ANOSIM results are shown in Table 1. All values are presented using Global R statistic values and level of significance (*p*-value). Large R-values (close to +1 or −1) indicate greater dissimilarity between the two groups. Overall, in the ANOSIM test, genotype factors showed R values lower than those of year vs. year for most nutritional components, indicating a lower contribution of genetic factors than the year effect. Cultivation year exhibited the greatest influence on the composition of proximates (R = 0.702, *p* = 0.001) and amino acids (R = 0.690, *p* = 0.001), followed by non-Bt rice vs. Ref, and lastly by Bt rice vs. Ref. In addition, vitamins and minerals were different between the plant-growing years (R = 0.543, *p* = 0.001 for vitamins; R = 0.402, *p* = 0.001 for minerals). However, cultivation sites contributed to nutritional compositions to a lesser extent compared with cultivation years. There were significant differences in proximates (R = 0.296, *p* =0.019), fatty acids (R = 0.146, *p* = 0.022), and minerals (R = 0.15, *p* = 0.016) of Bt rice vs. non-Bt rice. R-values of Bt rice vs. non-Bt rice were lower for proximates and minerals than those of Bt rice vs. Ref, indicating that difference between Bt rice and non-Bt rice is smaller than that of between Bt rice and Ref. For example, non-Bt rice vs. Ref exhibited the greatest impact on the composition of proximates (R = 0.431, *p* = .001), followed by Bt-rice vs. Ref (R = 0.419, *p* = 0.001) and lastly by Bt rice vs. non-Bt rice (R = 0.296, *p* = 0.001). There was no significant difference in antinutrients by the genotype factors. Bt rice vs. non-Bt rice did not exhibit significant differences (*p* = 0.05) in amino acids, vitamins, and antinutrients. R-value of fatty acids (R = 0.146, *p* = 0.022) of Bt rice vs. non-Bt rice was slightly lower than that of Bt rice vs. Ref (R = 0.117, *p* =0.049).

**Table 1.** Analysis of similarities (ANOSIM) results between two effect factors in rice composition

Nutrition category	R^a^	Year vs. Year^e^	Site^c^ vs. Site	Bt rice vs. non-Bt rice^d^	Bt rice vs. Ref^e^	non-Bt vs. Ref
	*p*^b^					
**Proximates**	R	0.702	0.239	0.296	0.419	0.431
	*p*	0.001	0.001	0.019	0.001	0.001
**Amino acids**	R	0.690	0.232	0.161	0.405	0.299
	*p*	0.001	0.001	0.054	0.001	0.001
**Fatty acids**	R	0.218	0.065	0.146	0.117	−0.068
	*p*	0.001	0.017	0.022	0.049	0.877
**Minerals**	R	0.402	0.220	0.150	0.429	0.210
	*p*	0.001	0.001	0.016	0.001	0.003
**Vitamins**	R	0.543	0.233	0.080	0.093	0.160
	*p*	0.001	0.001	0.342	0.066	0.008
**Anti-nutrients**	R	0.351	0.138	−0.046	0.018	0.096
	*p*	0.001	0.001	0.826	0.67	0.531
**Mean**	R	0.484	0.188	0.166	0.247	0.239

Plant cultivation year, cultivation site, genetic modification, and genotype effects on the nutritional components in rice were calculated using Bray-Curtis similarity matrices (ANOSIM), based on transformed abundance data. ^a^ R: Sample statistic (Global R); ^b^ *p*: significance level of sample statistic; ^c^ Site: Suwon and Jeonju (South Korea cultivation site); ^d^ Bt rice: insect-resistant rice; non-Bt rice: direct comparator; ^e^ Ref: commercial reference rice cultivars

### Variance Analysis Using PERMANOVA and SIMPER

PERMANOVA and pairwise comparisons were used to determine whether nutritional components were significantly affected by existing environmental and genotypic factors, as well as by interactions among these factors. The variation (COV) component in PERMANOVA was a value that represented the effect of each factor on nutritional variance; the larger the COV value, the greater the effect of a particular factor on the differences observed among the study groups. In contrast to ANOVA, which relies on normal distributions, PERMANOVA is a nonparametric analysis of variance using distances between samples and uses a permutation method to test the hypothesis. PERMANOVA results of *p* (perm) values and COV are shown in Table 2. A significantly higher COV value (*p* < 0.01) indicates a greater degree of influence of each factor.

**Table 2.** Results of the PERMANOVA analysis for the nutrition categories of rice grains, using plant cultivation year, cultivation site, genotype, and their interaction as the source of variation

Nutrition category	Factor	df	Pseudo-F	*p* (perm)	Components of variation (COV)
**Proximates**	Y	1	188.600	0.001	5.722
	S	1	0.408	0.755	−0.018
	G	2	31.390	0.001	1.236
	G × S × Y	2	13.206	0.001	1.986
**Amino acids**	Y	1	17.729	0.001	0.195.
	S	1	96.287	0.001	0.034
	G	2	68.795	0.001	0.185
	G × S × Y	2	30.131	0.001	0.317
**Fatty acids**	Y	1	6.772	0.002	0.205
	S	1	1.350	0.240	0.008
	G	2	7.936	0.001	0.128
	G × S × Y	2	0.674	0.638	−0.039
**Minerals**	Y	1	14.470	0.001	0.113
	S	1	78.356	0.001	0.651
	G	2	14.751	0.001	0.154
	G × S × Y	2	14.527	0.001	0.607
**Vitamins**	Y	1	23.812	0.003	3.299
	S	1	9.241	0.001	0.224
	G	2	1.310	0.001	0.816
	G × S × Y	2	0.694	0.001	1.092
**Antinutrients**	Y	1	23.812	0.001	0.196
	S	1	9.241	0.005	0.071
	G	2	1.310	0.274	0.004
	G × S × Y	2	0.694	0.496	−0.014

df, degrees of freedom; Pseudo-F; F value by permutation; *p* (perm), *p*-value by permutation;

Y, plant cultivation year; S, plant cultivation site; G, genotype (Bt rice, non-Bt rice, and reference rice); G × S × Y, interaction of genotype, site, and year.

The factor that most significantly influenced natural variability in proximates which had the highest COV value was cultivation year (COV = 5.722), followed by the G × S × Y effect (COV = 1.986) and then genotype (COV = 1.236). For amino acids, the G × S × Y effect (COV = 0.317) had the most influence, followed by cultivation year (COV = 0.195) and then genotype (COV = 0.185). Cultivation year (COV = 0.205) and genotype (COV = 0.128) had significant roles for fatty acids. With regards to the mineral contents, there was a significant association with cultivation site (COV = 0.651) and the G × S × Y effect (COV = 0.607). Cultivation year (COV = 0.113) and genotype (COV = 0.154) were also had significant influences on mineral. In agreement with our data, the variability of rice mineral compositions was attributable to the plant cultivation site rather than to the variety or cultivation year.^50^ Cultivation year (COV = 3.299) played the largest role for vitamin. The G × S × Y effect (COV = 1.092) also influenced vitamin. Cultivation year (COV = 0.196) was the most significant factor accounting for the levels of antinutrients (Table 2).

**Figure 2.**
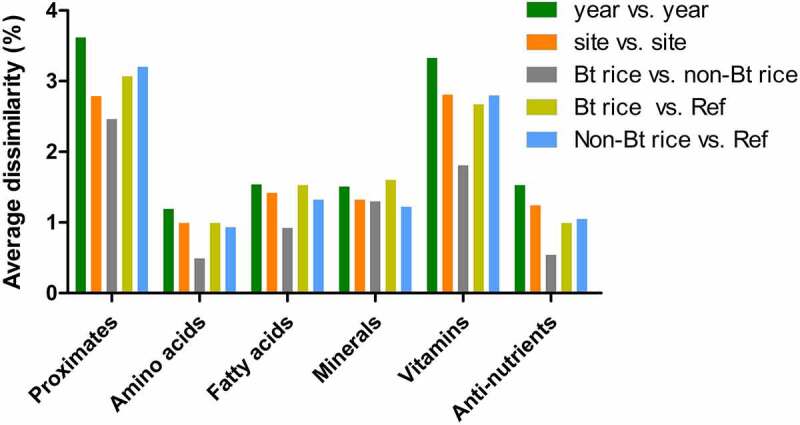
Average dissimilarity in the composition of proximates (a), amino acids (b), lipid acids (c), minerals (d), vitamins (e), and antinutrients (f) among cultivation factors and among genotype factors by SIMPER analysis

**Figure 3.**
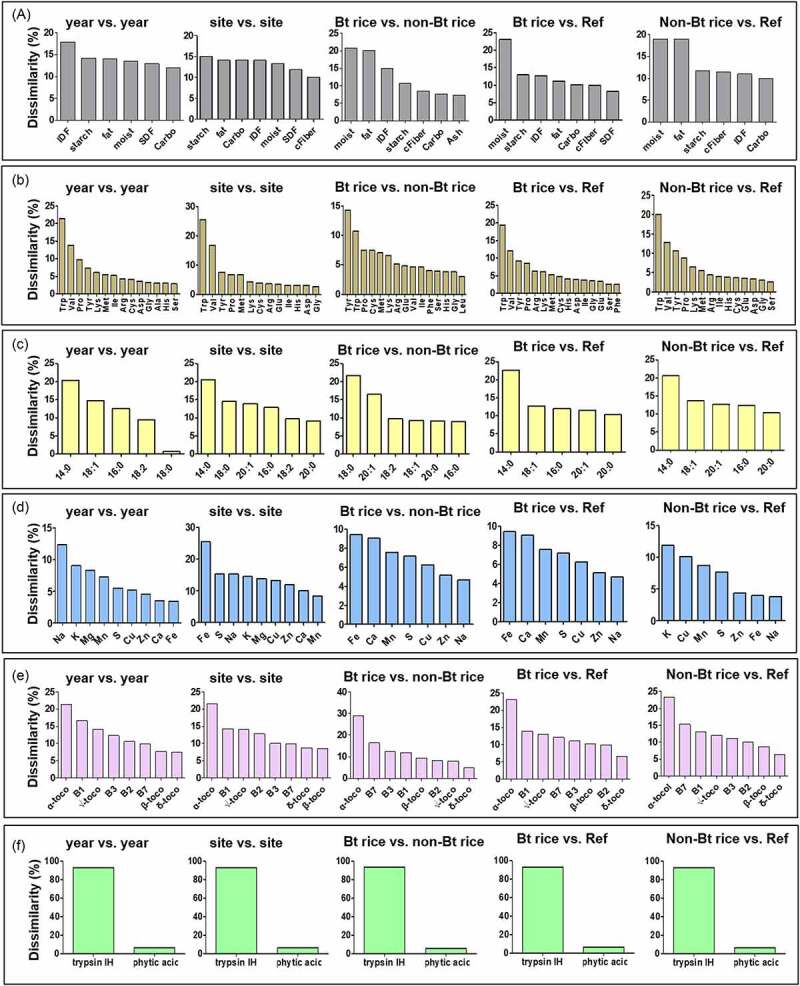
Results of SIMPER comparison among cultivation factors and among genotype factors for all rice components. SIMPER analysis was operated using a two-way crossed layout, opting for the Bray-Curtis dissimilarity matrix, and setting the cutoff at 80%. Abbreviation: Carbo, carbohydrate; Trypsin IH, Trypsin inhibitor

The average dissimilarity between pairs of study groups and the relative contribution of each analyte to the observed dissimilarity was determined by the SIMPER test. The average dissimilarity in study groups for the nutrient category is shown in Fig. 2. The analytes of each nutrient category were presented in the order of contribution significance to the average dissimilarity between study groups (Fig. 3, Table S3 of the Supporting Information). Overall, the comparison between cultivation years presented the most significant dissimilarity in nutrient categories (Fig. 2). By contrast, the lowest level of dissimilarity was detected in the pairwise comparison between Bt rice and non-Bt rice. These trends were consistent with the results of ANOSIM (Table 1), demonstrating that the most significant effect of cultivation year and the lowest effect of transgene insertion on the composition. Proximates and vitamins showed a more significant average dissimilarity than amino acids, fatty acids, minerals, and antinutrients (Fig. 2, Table S3 of the Supporting Information). The average dissimilarity in proximates between the cultivation years was approximately 3.62% (Fig. 2, Table S3 of the Supporting Information). IDF was the highest contributor to the differences in proximates observed between cultivation years (17.92%) (Fig. 3a). This result is in accordance with the percentage variability analysis, which showed a significant effect of the cultivation year on the IDF level (Fig. 1). Comparisons between Bt rice and non-Bt rice revealed that the most significant dissimilarity in the proximate results was contributed by moisture (20.83%) (Fig. 3a). However, the average dissimilarity between Bt rice and non-Bt rice was approximately 2.46%, which was the lowest value obtained among study groups (Table S3 of the Supporting Information). Notably, moisture contributed the most to the dissimilarity in the pairwise comparisons of genotype study groups. This result is in line with the statistical differences between Bt rice and non-Bt rice moisture content (Table S2). Percentage variability analysis determined that carbohydrate was influenced mainly by the G × S × Y effect, followed by site and cultivation year (Fig. 1). However, the contribution of carbohydrates to dissimilarity in the pairwise comparisons of study groups was not significant (Fig. 3a).

The SIMPER results for amino acids revealed that tryptophan, valine, and tyrosine mainly determined dissimilarities among all study groups (Fig. 3b, Table S3 of the Supporting Information). The lowest average amino acid dissimilarity was 0.49% for the Bt rice vs. non-Bt rice comparison (Fig. 2). The dissimilarity for fatty acids was, on average, generated mainly by myristate (14:0) and oleic acid (18:1) in all study groups (Fig. 3c). For minerals, the extent of the dissimilarity between pairwise comparisons was similar among the study groups (Fig. 2). The primary vitamin component contributing to the dissimilarity among the study groups was α-tocopherol (Fig. 3e). For antinutrients, the highest average dissimilarity was found between cultivation years (1.53%), while the lowest one was observed between Bt rice vs. non-Bt rice (0.54%) (Fig. 2). Trypsin inhibitor was the main antinutrient component contributing to the dissimilarity among all groups (fig. 3f). The significant differences between compared pairs identified by ANOSIM (Table 1) were confirmed with SIMPER analysis (Fig. 2, Table S3 of the Supporting Information), thereby identifying the nutrient components that primarily contributed to the differences observed (Fig. 3).

In the present study, Bt rice exhibited statistically significant differences compared with non-Bt rice (*p* < 0.05) in 19 components from Suwon and 32 components from Jeonju for either of the two years or both (Table S2 of the Supporting Information). However, the mean values of these components in Bt rice were within the reference or OECD ranges, indicating the compositional equivalence of Bt rice and conventional comparators. The impact of both genetic and environmental factors on nutritional variability and degree of similarity/dissimilarity among the study groups in rice grains was demonstrated by multivariate analysis. The content of rice grain components was more influenced by the cultivation year than by other factors. Results obtained from ANOSIM and SIMPER analyses were complementary, explaining the factors responsible for the differences among study groups, and thus provided a high degree of reliability. In addition, SIMPER analysis identified which nutrient components primarily contributed to the differences observed. We showed that the application of ANOSIM, PERMANOVA, and SIMPER is of value in understanding the difference and similarity of Bt rice against conventional rice in the context of natural variation. These approaches can be effectively applied in future safety assessments of crops developed by new breeding technologies, as well as GM crops.

## Supplementary Material

Supplemental Material
