# Supplementary figures and images for: Compositional equivalence assessment of insect-resistant genetically modified rice using multiple statistical analyses
Source: GM Crops Food. 2021 Mar 1;12(1):303–14. doi: 10.1080/21645698.2021.1893624 (PMC7928020; doi:10.1080/21645698.2021.1893624)

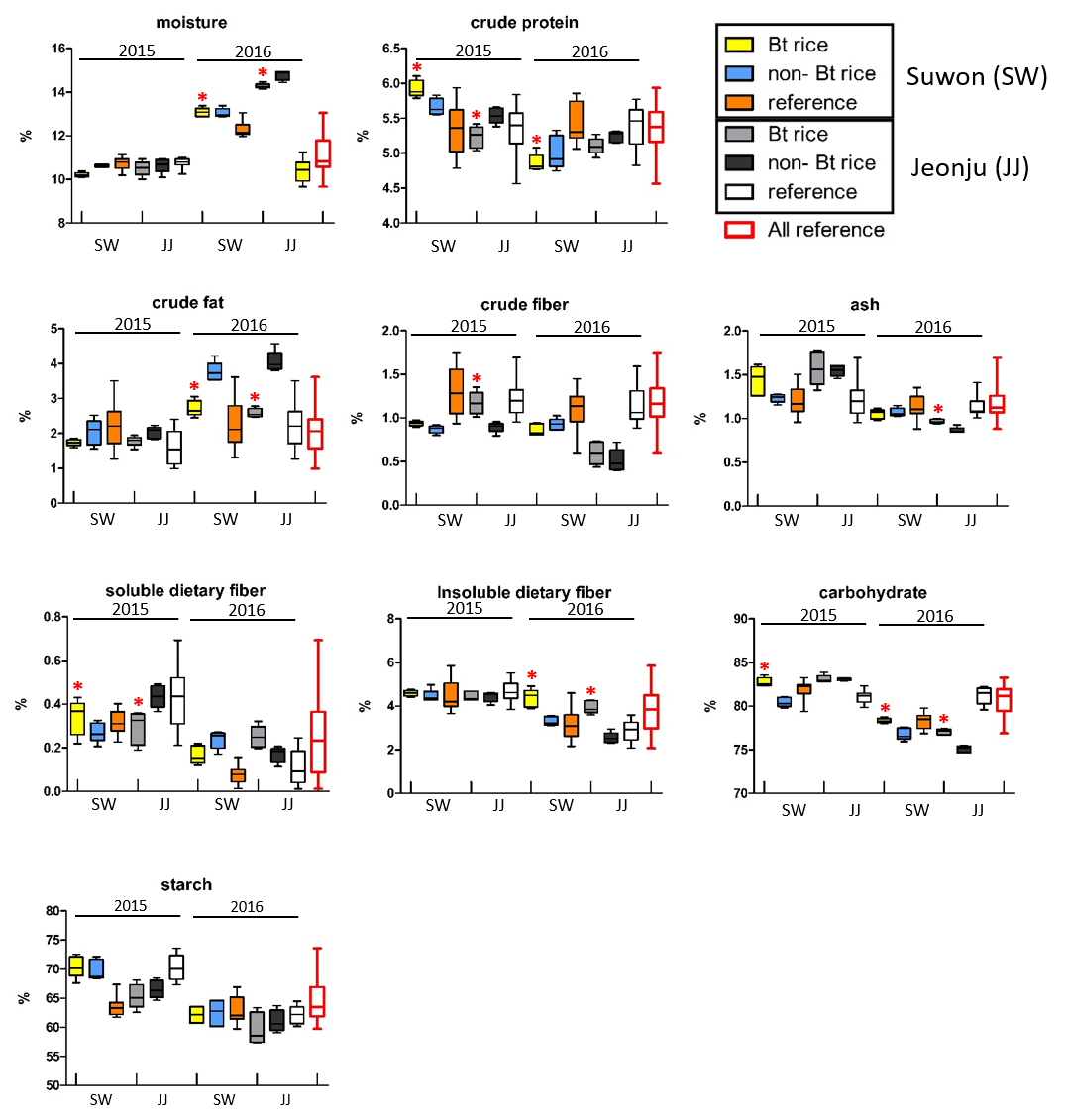

Supplement: Supplemental Material [file KGMC_A_1893624_SM8961.zip › Figure S1.png]

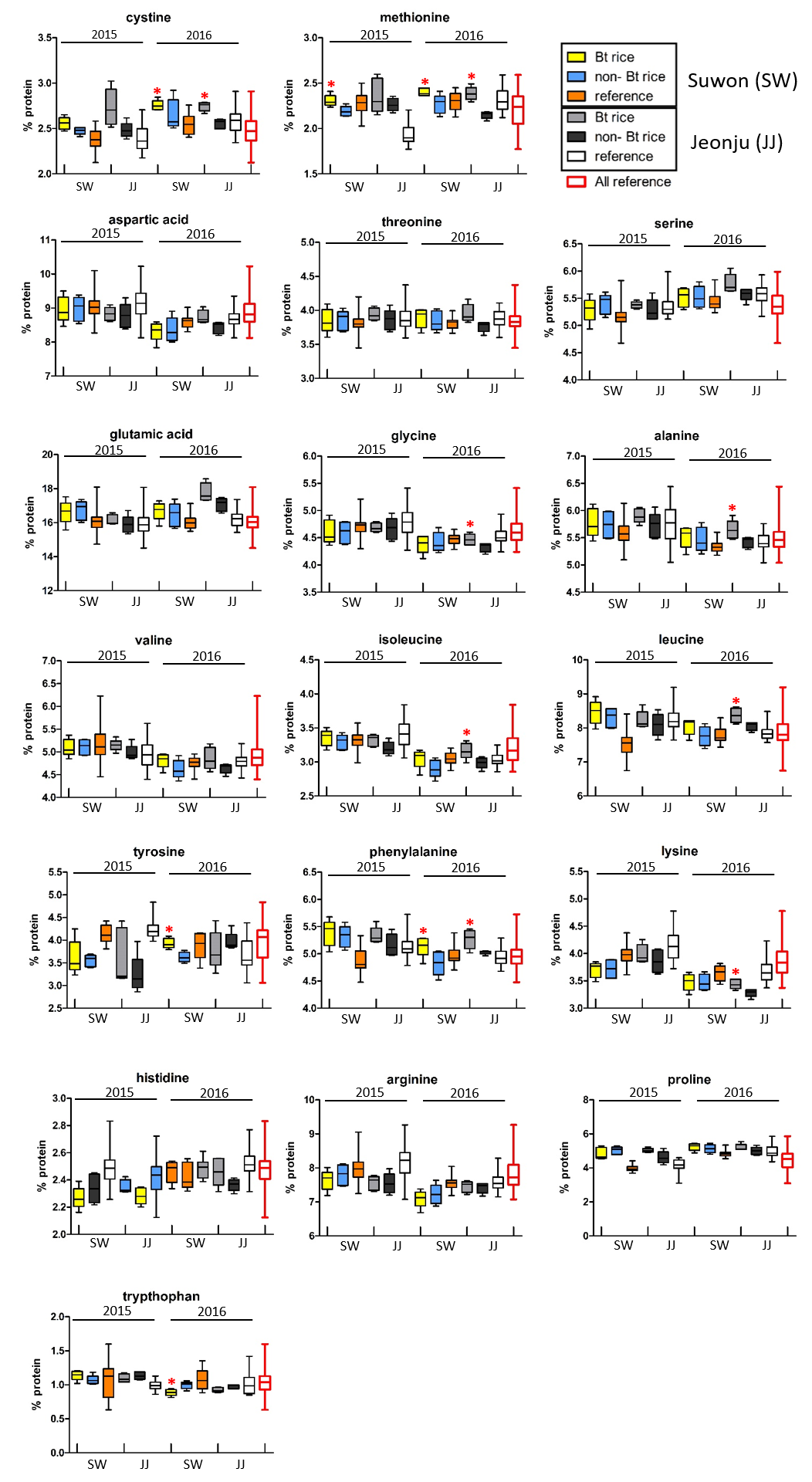

Supplement: Supplemental Material [file KGMC_A_1893624_SM8961.zip › Figure S2.png]

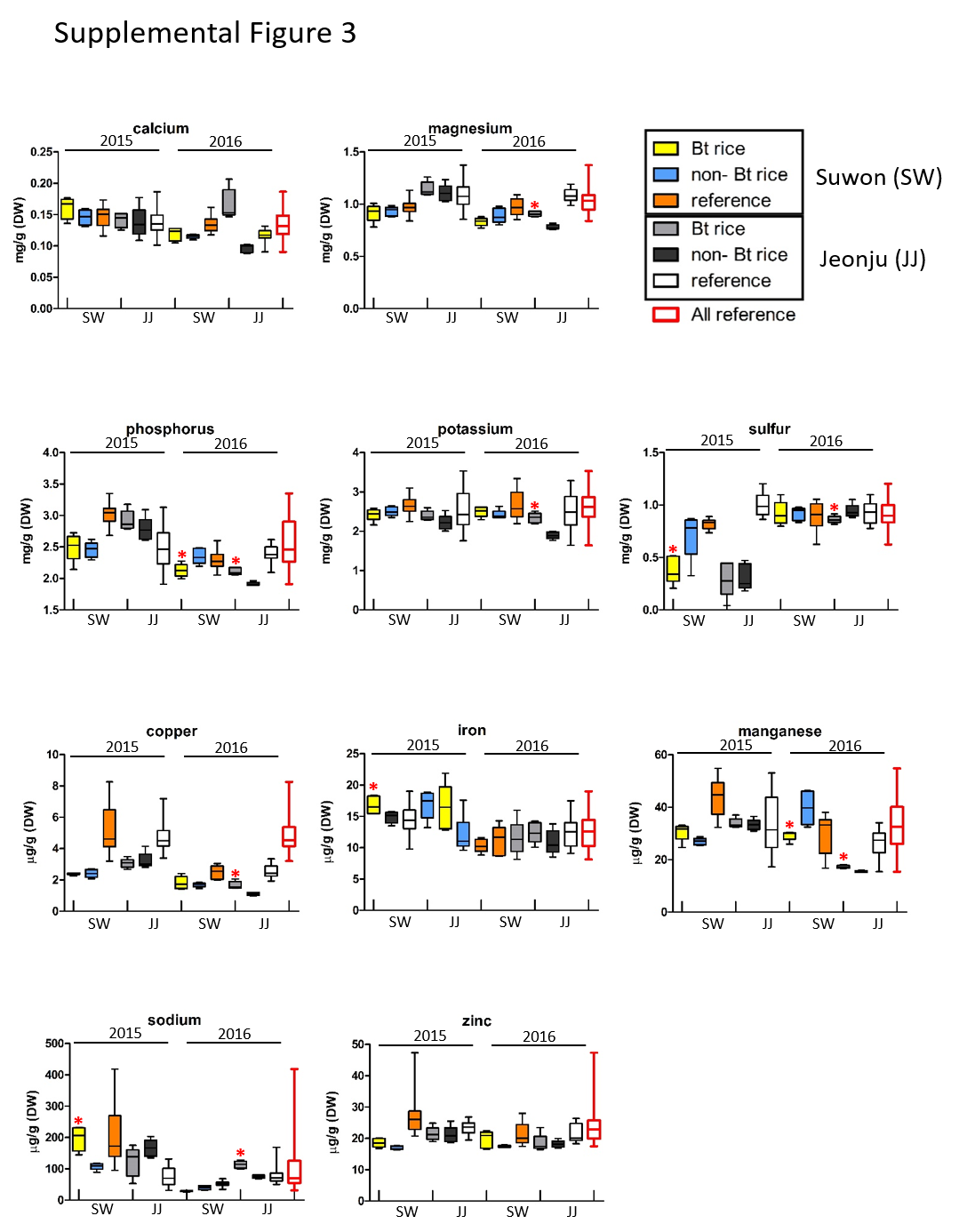

Supplement: Supplemental Material [file KGMC_A_1893624_SM8961.zip › Figure S3.png]

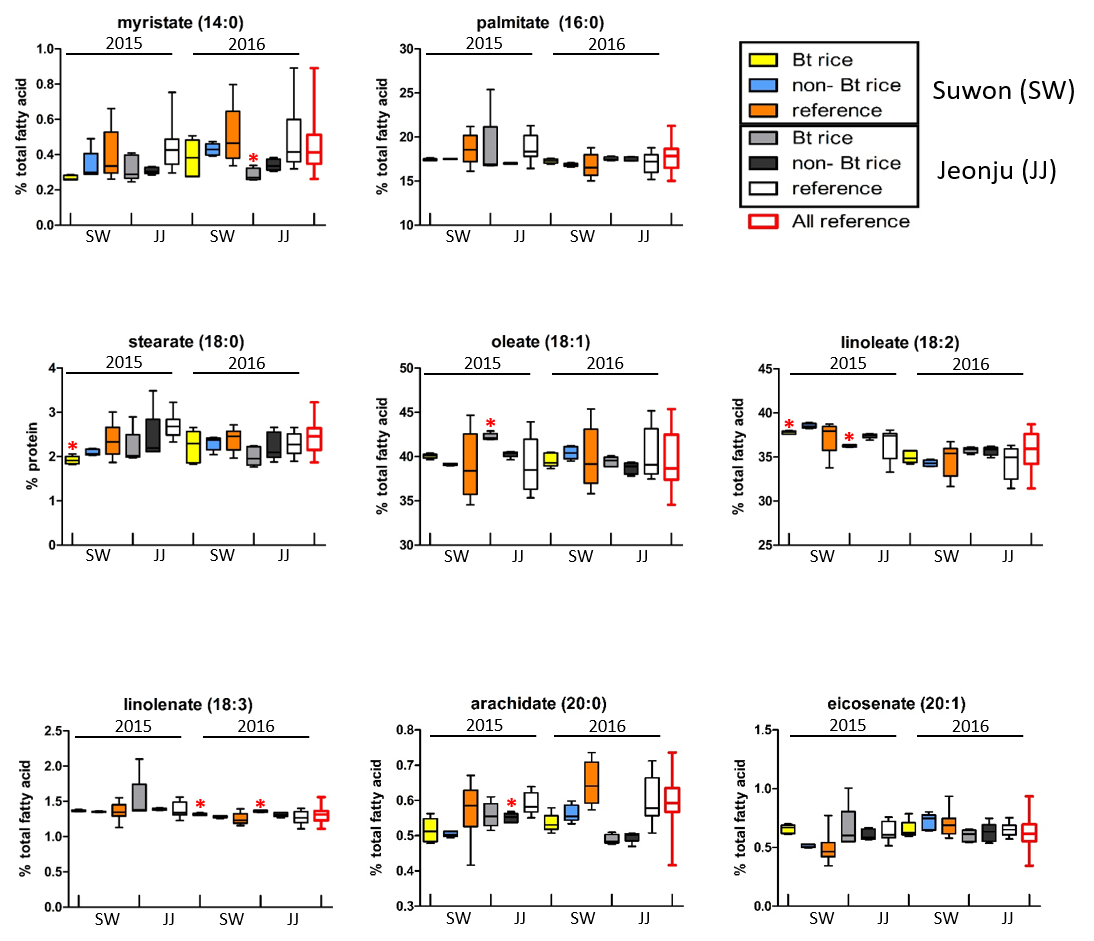

Supplement: Supplemental Material [file KGMC_A_1893624_SM8961.zip › Figure S4.png]

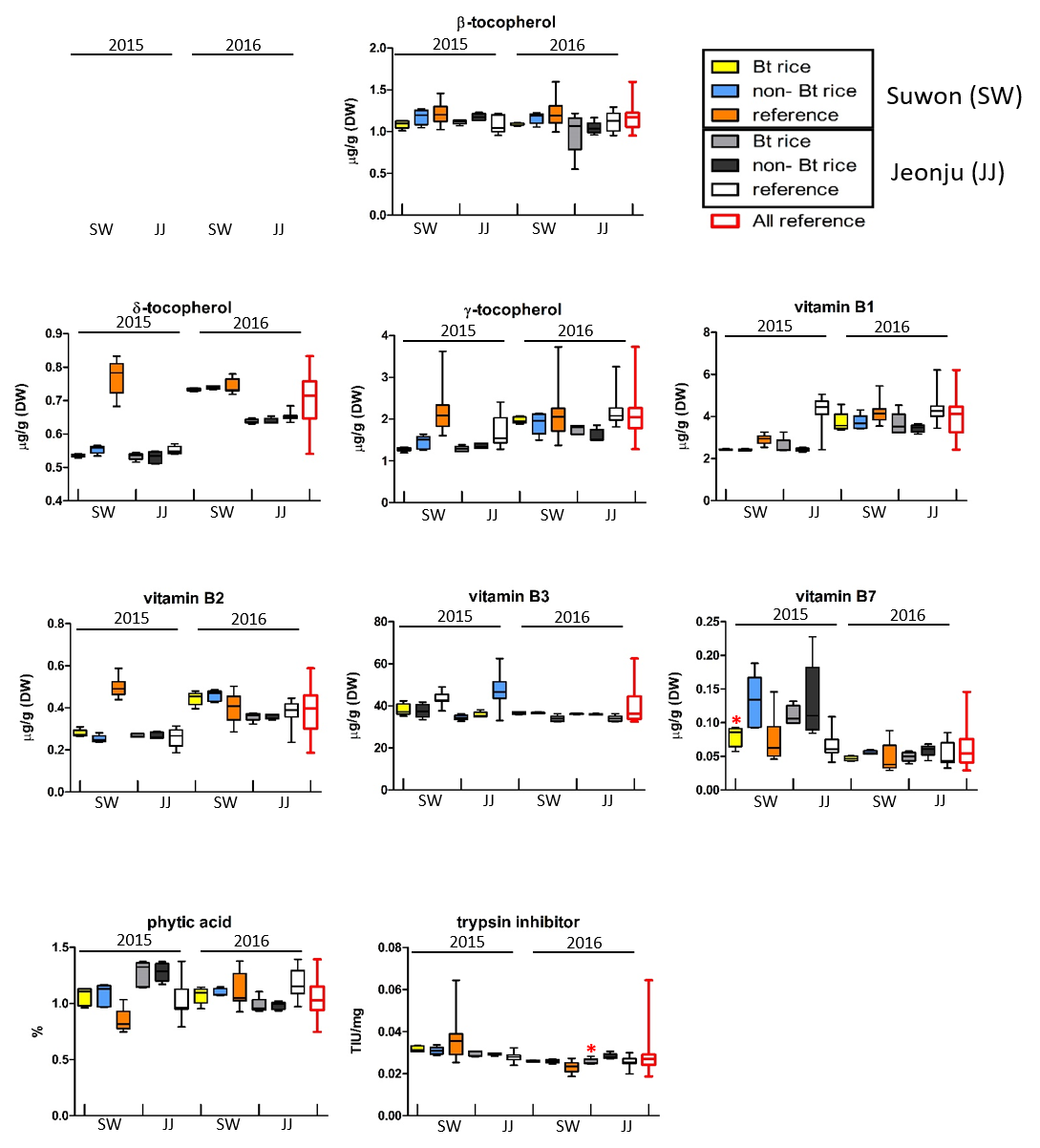

Supplement: Supplemental Material [file KGMC_A_1893624_SM8961.zip › Figure S5.png]
